# Quasi-Crystal Metasurface for Simultaneous Half- and Quarter-Wave Plate Operation

**DOI:** 10.1038/s41598-018-34142-y

**Published:** 2018-10-24

**Authors:** Meraj-E- Mustafa, Muhammad Amin, Omar Siddiqui, Farooq A. Tahir

**Affiliations:** 1 Research Institute for Microwave and Millimeter-wave Studies, National University of Sciences and Technology, Islamabad, Pakistan; 2 College of Engineering, Taibah University, P. O. Box 344, Madinah, Saudi Arabia

## Abstract

We present a quasi-crystal metasurface that can simultaneously work as efficient cross-polarizer and circular polarizer for wide range of frequencies. The quasi-crystal technique benefits from individual resonant response of anisotropic patch and the coupled response due to periodic perturbations in the square lattice. It is shown that quasi-crystals offer broadband response for cross-polarization as well as high efficiency circular-polarization conversion of reflected fields. The quasi-crystal metasurface achieves cross-polarization (above −3 dB) for two broad frequency bands between 10.28–15.50 GHz and 16.21–18.80 GHz. Furthermore, the proposed metasurface can simultaneously work as high efficiency circular-polarizer from 10.15–10.27 GHz and 15.51–16.20 GHz. The metasurface design is also optimized to suppress co-polarization below −10 dB between 10.5–15.5 GHz. This metasurface can find potential applications in reflector antennas, imaging microscopy, remote sensing, and control of radar cross-section etc.

## Introduction

Polarization state nature of electromagnetic (EM) waves influence the wave-matter interaction. Many applications such as optical sensing, satellite communications, reduction in radar cross section and contrast imaging microscopy are realized by controlling the orientation of polarization state of waves. Previously, traditional methods such as Faraday effect, optical activity of crystals and birefringence in anisotropic crystals are utilized for polarization state control of EM wave. However, such methods have the inherent disadvantage of narrow bandwidth and bulkier volume at low frequencies.

Recently, polarization property of EM wave is manipulated by employing certain type of planar metasurfaces^1–3^. Realization of such all-wave technology rely on efficiently controlling the flow of photons. Classical bandgap technology is used to demonstrate the control of wave propagation at sub-wavelength scale^4–9^. Such bandgap can be realized by building periodic dielectric materials in one, two, or three dimensions known as photonic crystals^10^. Recently, regular photonic crystals based metasurfaces are modified to form quasi-crystals that offer advantages such as higher point group symmetry and broadband operation^11–13^.

The bandgap mechanism of quasi-crystals is also translated to the resonant interference between radiating elements in structured metallic metasurfaces. The metallic two-dimensional quasi-periodic unit-cells are arranged to control wave propagation at sub-wavelength scale. The collective radiation characteristics of such quasi-crystal resonators depends upon the individual response of metallic radiating elements and mode coupling among them. In this context, planar metallic structures with certain periodicity are analyzed for light-harvesting and increasing light absorption etc.^14–19^. Quasi-crystal structures are now utilized to efficiently control and trap light at nanoscale for solar cell applications^20–22^.

Anisotropic metasurfaces are generally used for polarization conversion of EM waves^23–32^. The chiral metasurfaces achieve linear and circular polarization conversion in microwave^33–39^, THz^40–43^, and visible^44–46^ frequency regimes. In recent years, reconfigurable metasurfaces have gained special interest because of their wide band polarization conversion and tunable operation^47–49^. However, the reconfigurability has its own multiple drawbacks including fabrication complexity, increased cost and complex functionality. In general, broadband high efficiency polarization conversion is achieved by stacking multi-resonators layers upon each other. These resonances at different frequencies are coupled together to give a broadband response^50–52^. Such broadband response is realized at the cost of complex fabrication process and therefore it is incompatible with planar optical and microwave devices.

Planar metasurfaces are realized to achieve narrow-band half-wave and quarter-wave plate operation using cut-wire-pair structures at THz frequencies^53^. Improved anisotropic structures are realized to enable broadband half- and quarter- wave plate operations for infrared and THz frequencies^54,55^. It is worth mentioning that narrow-band half- and quarter- wave plate operation is recently reported at microwave frequencies^56^. However, realizing broadband chirality for reflected fields only using single layer of ultrathin metasurface is desirable.

In this paper, we present a design of single layer quasi-crystal metasurface to achieve broadband chiral response (i.e., simultaneous half- and quarter- wave plate operation) for reflected fields. The quasi-crystal design consists of super cell made of variable sized anisotropic metallic patches arranged in square lattice. This variation in size of metallic patches inside super cell introduce disorder that is periodically repeated in the lattice. The proposed metasurface acts as half-wave plate (HWP) to convert linear and circular polarized incident waves to their respective cross-polarizations for multiple broadband frequency bands. The cross-polarization-conversion efficiency remains above 90% for broad range of frequency by optimizing the geometric parameters of the supercell. Additionally, the structure behaves as an efficient quarter-wave plate (QWP) capable to transform linear to circular and circular to linear polarizations at multiple frequency bands. Such a broadband ultrathin metasurface will help miniaturize the size of microwave and optical system and can be used in broad range of applications.

## Results

### Design of Quasi-Crystal Metasurface

**Figure 1:**
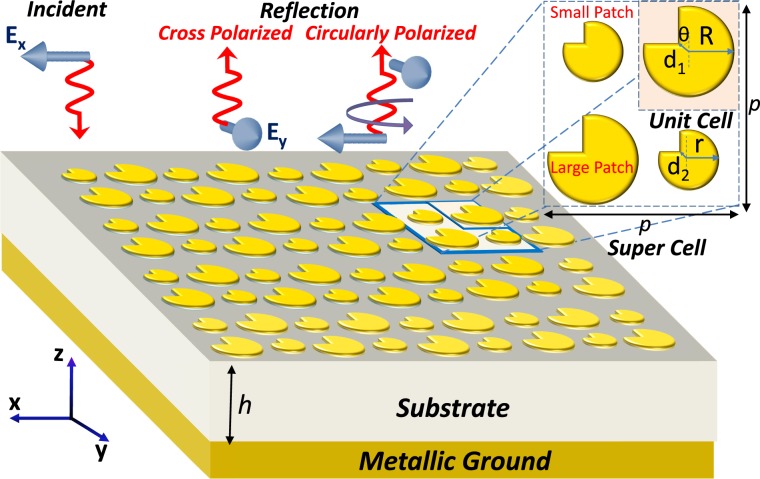
Schematic illustration of the proposed quasi-crystal metasurface. Inset shows the front view of individual unit cell and periodic array of super cell.

The quasi-crystal metasurface design consists of square lattice of anisotropic metallic patches. The basic unit cell is composed of anisotropic circular patch as shown in Fig. 1. The radius of each circular patch is defined as *R* having a square cut at its top left corner (i.e., *θ* = 45°) represented by diagonal distance *d*. The periodicity of unit cell is *p*/2, as shown in Fig. 1. The super cell is a collection of multiple (four) unit cells that incorporate larger area, as shown in Fig. 1. The period of super cell is defined as *p*. The metasurface is supported by Rogers 5880 substrate with relative permittivity of 2.2 and loss tangent of 9 × 10^−4^. Anisotropic patches and ground plane are made of copper with conductivity of 5.8 × 10^7^ S/m and thickness of 17 *μ*m. The geometrical parameters defined in Fig. 1 for anisotropic patch are *θ* = 45° and *d*_1,2_ = 0.707 mm unless stated otherwise. The period of super- and unit- cell is *p* = 20 mm and *p*/2 = 10 mm respectively.

**Figure 2:**
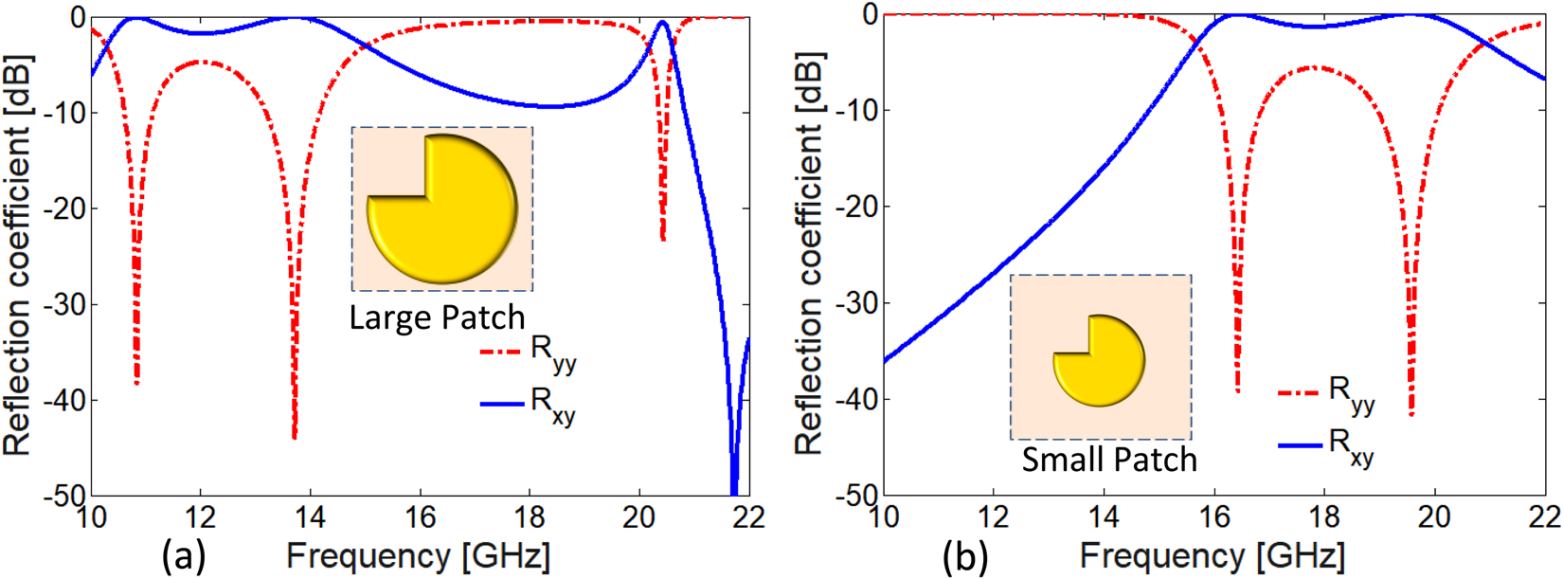
(**a**) Co- and cross-polarized reflection coefficient of unit cell with large patch (**b**) Co- and cross-polarized reflection coefficient of unit cell with small patch.

If we look at the design evolution of the super cell of quasi crystal metasurface, initially a complete circular patch was simulated and analyzed. Because of the isotropic nature of the circular patch, no cross-polarization was observed. Anisotropy was then introduced by putting a square cut at the top left corner of the circular element as shown in the inset of Fig. 2(a). Owing to this anisotropy, the desired HWP and QWP operation is achieved at different frequency bands. Then, through full-wave simulations the parameter “d” and substrate thickness “h” are optimized to get maximum polarization conversion response. Similarly, another similar but relatively small patch is optimized using same parameters to achieve cross-polarization response relatively at higher frequencies. Finally, both anisotropic elements are diagonally placed in a square lattice making a supercell of size 2 × 2. This supercell is then briefly optimized to refine simultaneous HWP and QWP operations over multiple and wide frequency bands.

### Half-Wave Plate Operation

HWP or cross-polarizer converts linearly or circularly polarized incident waves into its corresponding cross-polarized components upon reflection from the metasurface. The relation between linearly polarized incident and reflected fields is established with Jones reflection matrix in Cartesian basis. We consider *E*_*xi*_ and *E*_*yi*_ as incident electric field components in *x* and *y* directions respectively. Similarly, consider *E*_*xr*_ and *E*_*yr*_ as reflected electric field components in *x* and *y* directions respectively.

1 $$(\begin{array}{c}{E}_{xr}\\ {E}_{yr}\end{array})=(\begin{array}{cc}{R}_{xx} & {R}_{xy}\\ {R}_{yx} & {R}_{yy}\end{array})(\begin{array}{c}{E}_{xi}\\ {E}_{yi}\end{array})$$

Here, *R*_*xx*_ = |*E*_*xr*_|/|*E*_*xi*_| and *R*_*yy*_ = |*E*_*yr*_|/|*E*_*yi*_| are the co-polarized reflection coefficients for *x* and *y*- polarized incident waves respectively. While, *R*_*yx*_ = |*E*_*yr*_|/|*E*_*xi*_| and *R*_*xy*_ = |*E*_*xr*_|/|*E*_*yi*_| are the cross-polarized reflection coefficients for the *x*- and *y*- polarized incident wave respectively. Ideally, the cross-polarizer suppresses co-polarized components i.e., *R*_*xx*_, *R*_*yy*_ = 0 and supports near unity cross-polarized components i.e., *R*_*xy*_, *R*_*yx*_ = 1.

### Disorder less Crystal Metasurface

First, we investigate the response of identical disorder less crystals without perturbations. The anisotropy of patches with identical unit cells (i.e., *R* = *r* and *d*_1_ = *d*_2_) makes the unit cells inside super cell isomorphic.

The co- and cross- polarized reflection spectrum for the two different sized larger and smaller patch are shown in Fig. 2(a,b) respectively. As expected the smaller patch of radius *r* = 2.85 mm is resonating at higher frequencies compared to large patch of radius *R* = 4.2 mm. For larger patch of radius *R* = 4.2 mm, the metasurface is resonant at lower frequencies i.e., 10.8 GHz, 13.7 GHz and 20.4 GHz. At resonance frequencies the co-polarized reflection coefficient for large patch reduces to −38 dB, −44 dB and −23 dB correspondingly increasing cross-polarization component. Similarly, for small patch of radius *r* = 2.85 mm, the metasurface is resonating at 16.4 GHz and 19.5 GHz, the co-polarized reflection coefficient *R*_*yy*_ for small patch reaches −39 dB and −41 dB, see Fig. 2(b).

Hence, the incident *x*-polarized wave is efficiently converted to *y*- polarized wave and vice versa at the resonance frequencies. Considering 3 dB bandwidth for cross-polarization, two distinct non-overlapping bands i.e., 10.3–15 GHz and 15.6–20 GHz are supported by large and small patch metasurfaces respectively. It will be interesting to benefit from the supported resonance bands from these small and large sized patches simultaneously.

### Periodically Disordered Quasi-Crystal Metasurface

Periodic disorder can be incorporated in the lattice by varying the size of anisotropic patches. The modified periodic array each containing four patches/unit cells constitute the super cell, see the inset in Fig. 1. As shown in the previous section that the resonances are sensitive to radius *R* of the patches, the radius is varied alternatively for each patch to periodically introduce such disorders. The small patches of radius *r* = 2.85 mm are situated along the diagonal, whereas the large patches of radius *R* = 4.2 mm are located along anti-diagonal. Hence, the variation in size of patches adds periodic resonant perturbations in both *x*- and *y*- directions. The metallic quasi-crystals under square lattice (i.e., periodic array of 2 × 2 super cell size) offer unique polarization insensitive characteristics under normal incidence as identical patches are placed along the diagonal of super cell^22^.

### Quasi-Crystal Metasurface as Broadband Cross Polarizer

**Figure 3:**
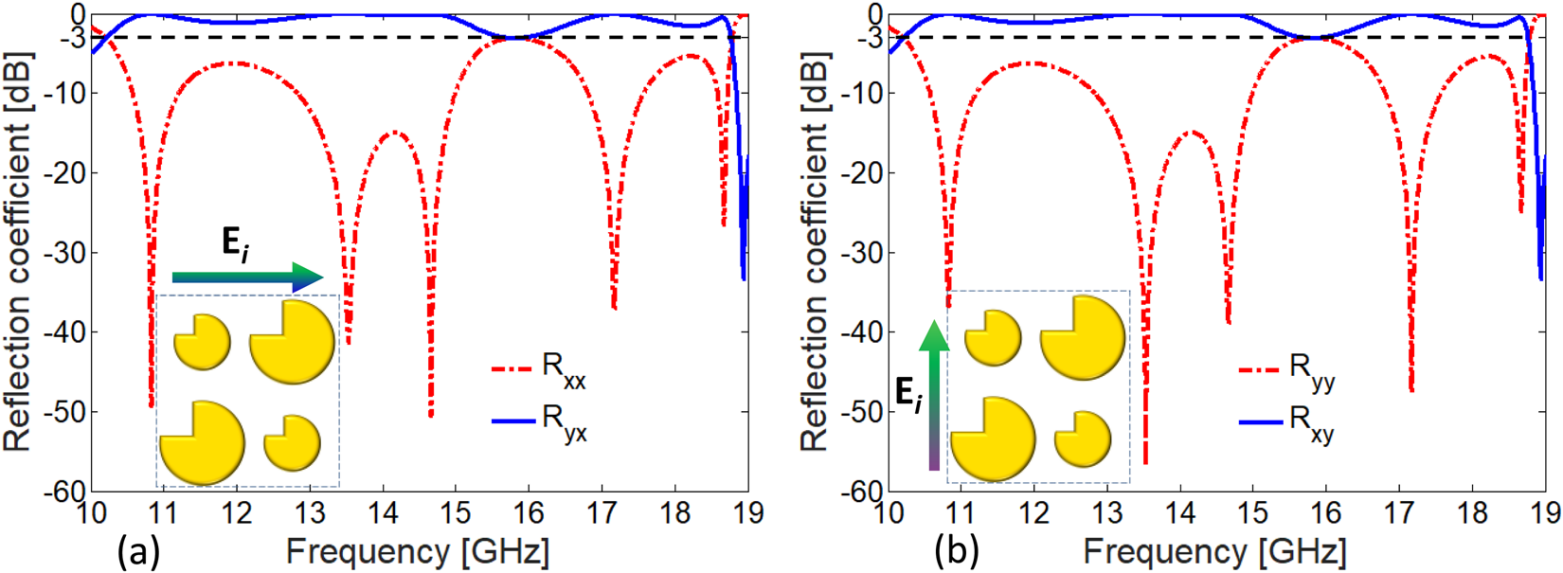
(**a**) Co- and cross-polarized reflection components for super cell optimized for maximum bandwidth of near ideal cross-polarization not less than −3 dB for (**a**) *x*- polarized (**b**) *y*- polarized incident wave.

It is worth mentioning that the resonances are highly sensitive to geometric parameters of quasi-crystal patches and the substrate height *h*, shown in Fig. 1. To overcome the sensitivity due to geometric parameters, the bandwidth of cross-polarization components (*R*_*xy*_ and *R*_*yx*_) are optimized for a minimum of −3 dB level. The optimized parameters are *d* = 0.707 mm, *θ* = 45°, *h* = 1.58 mm, and *R* = 4.2 mm/2.85 mm for large and small patch respectively. Figure 3(a,b) shows reflection spectrum for anisotropic quasi-crystal structure for both *x*- and *y*- polarized incident waves. Half wave plate operation is achieved for two broad frequency bands between 10.28–15.50 GHz and 16.21–18.80 GHz under normal incidence as shown in Fig. 3(a,b). It should be noted that the co-polarization coefficients *R*_*xx*_ and *R*_*yy*_ doesn’t exceed a level of −3 dB throughout the bandwidth. It should also be noted that the response of quasi-crystal metasurface is nearly polarization insensitive and the minor difference is due to asymmetric location of square cut with reference to composition of supercell. The proposed metasurface in Fig. 3(a,b) can also be simultaneously used as high efficiency circular polarizer as shown in the following section.

### Simultaneous Quarter-Wave Plate Operation

The proposed QWP or circular polarizer converts linearly polarized incident wave into circularly polarized wave and vice versa upon reflection from the metasurface. In order to analyze the relation between linearly polarized incident and circularly polarized reflected fields with corresponding Jones matrix in circular basis. Lets suppose *E*_*xi*_ and *E*_*yi*_ as incident electric field components in *x* and *y* directions respectively. Similarly, consider *E*_+*r*_ and *E*_−*r*_ as right-handed circular polarized (RHCP) and left-handed circular polarized (LHCP) reflected electric field components respectively.

2 $$(\begin{array}{c}{E}_{+r}\\ {E}_{-r}\end{array})=(\begin{array}{cc}{R}_{+x} & {R}_{+y}\\ {R}_{-x} & {R}_{-y}\end{array})(\begin{array}{c}{E}_{xi}\\ {E}_{yi}\end{array})={R}_{cl}(\begin{array}{c}{E}_{xi}\\ {E}_{yi}\end{array})$$

The elements in matrix *R*_*cl*_ can also be converted to Cartesian basis as follows.

3 $$(\begin{array}{c}{E}_{+r}\\ {E}_{-r}\end{array})=(\begin{array}{c}{E}_{xr}+j{E}_{yr}\\ {E}_{xr}-j{E}_{yr}\end{array})=\frac{1}{\sqrt{2}}(\begin{array}{cc}{R}_{xx}+i{R}_{yx} & {R}_{xy}+i{R}_{yy}\\ {R}_{xx}-i{R}_{yx} & {R}_{xy}-i{R}_{yy}\end{array})(\begin{array}{c}{E}_{xi}\\ {E}_{yi}\end{array})$$

The elements in Jones matrix *R*_*cl*_ demonstrate the ability of the structure to convert linearly polarized incident fields i.e., *E*_*xi*_ and *E*_*yi*_ to circularly polarized reflected fields i.e., *E*_+*r*_ and *E*_−*r*_. Here, subscripts “+” and “—” represents right-handed and left-handed waves respectively. Scaling factor of $1/\sqrt{2}$ normalizes the reflected electric field vector between 0–1 and also satisfies the passivity constraint.

It is clear from Eq. 3 that the condition to achieve circularly polarized wave, the reflected wave must contain both x- and y- polarized components, ideally having same magnitude (i.e., |*E*_*xr*_| = |*E*_*yr*_|) and phase difference of ±90° (i.e., ∠*E*_*xr*_ − ∠*E*_*yr*_ = ±*π*/2).

**Figure 4:**
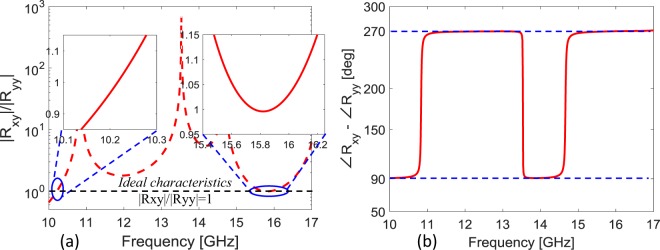
(**a**) Magnitude ratio of cross and co-polarized reflections. Inset shows the frequency region where metasurface exhibits ideal characteristics i.e., |*R*_*xy*_|/|*R*_*yy*_| = 1 necessary for circular polarization. (**b**) Phase difference between cross and co-polarized reflections for *y*- polarized incident wave.

The designed broadband cross-polarizer in Fig. 3(a,b) in section 2 simultaneously provides high efficiency quarter-wave plate characteristics at multiple frequency bands. The proposed metasurface is satisfying the criteria of quarter wave plate for both magnitude ratio bandwidth which is 0.85–1.15 and phase difference bandwidth which is 90° ± 5° or odd multiple of 90°. Figure 4(a) shows that reflection components *R*_*yy*_ and *R*_*xy*_ have nearly same magnitude (|*R*_*xy*_|/|*R*_*yy*_| ≈ 1) and a phase difference of (∠*R*_*xy*_ − ∠*R*_*yy*_ ≈ +270°) in the frequency range of 15.51–16.20 GHz, see Fig. 4(b). The condition for circular polarization is also met at narrow band around 10.15–10.27 GHz. It is worth mentioning that the two frequency bands offer near ideal circular polarization conversion. These ideal characteristics are attributed to negligible loss in the substrate, equal power distribution and ±90° phase difference between the two orthogonal reflection components.

The high efficiency of circular polarization conversion can be quantified by analyzing Polarization extinction ratio (PER). It is used to characterize the handedness and efficiency of the circularly polarized wave. High efficiency RHCP or LHCP is dominating in the structure at the particular frequency or in the frequency band depends upon the small or large PER. PER can be calculated as follows.

4 $${\rm{PER}}={\rm{20}}{\mathrm{log}}_{{\rm{10}}}(\frac{|{R}_{+y}|}{|{R}_{-y}|})$$

**Figure 5:**
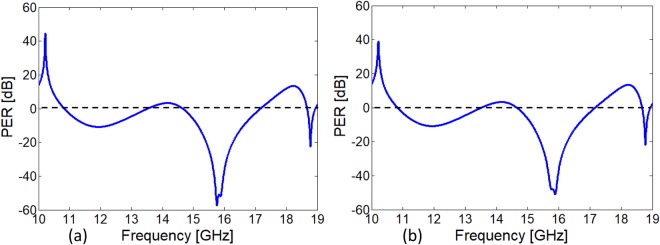
(**a**) Polarization extinction ratio for *x*-polarized incident wave (**b**) Polarization extinction ratio for *y*- polarized incident wave.

Here, *R*_+*y*_ and *R*_−*y*_ are the RHCP and LHCP conversion due to *y*- polarized incident electric field respectively. The proposed design also gives both the LHCP and RHCP at different frequencies. It can be noted from the calculated PER in Fig. 5(a,b) that RHCP wave is dominating at 10.23 GHz where the difference of magnitude between RHCP and LHCP is nearly 40 dB. While, LHCP is dominating in the frequency band of 15.51–16.20 GHz. Difference between the magnitudes of LHCP and RHCP reaches more than 50 dB in this band which means a pure circularly polarized wave is obtained. Additionally, LHCP resonance mode obtained at 18.76 GHz but the PER efficiency is limited to −20 dB at this frequency^38^.

### Angular Stability

**Figure 6:**
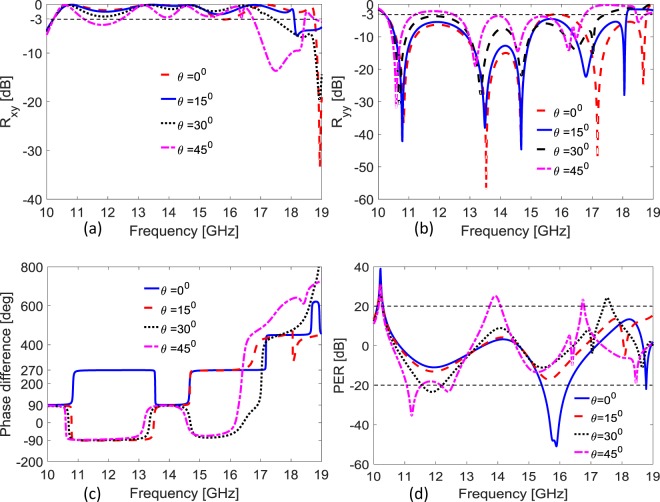
(**a**) Cross-polarized reflection coefficient at different incident angles (**b**) Co-polarized reflection coefficient at different incident angles (**c**) Phase difference between cross- and co-polarized reflections at different incident angles (**d**) PER at different angles of incoming wave.

The angular stability response of the quasi-crystal metasurface is also analyzed for incident angles ranging from 0° to 45°. It is clear from Fig. 6 that if the angle of incidence changes from 0° to 45°, the operating bandwidth of HWP operation remain almost stable upto 30° and QWP operation remain stable upto 45° for the first band (10.15–10.27 GHz) while for the 2nd band (15.51–16.20 GHz) no CP was observed above 0°.

An additional but interesting point is that if we look at 45° incidence in Fig. 6(d), we have achieved five different bands (10.15–10.27 GHz, 11.06–11.54 GHz, 12.16–12.54 GHz, 13.73–14.07 GHz and 16.70–16.77 GHz) where complete CP operation exists, this is only for 45° incidence with respect to 20 dB PER criterion for QWP operation^38^.

### Design of High Efficiency Cross Polarizer

**Figure 7:**
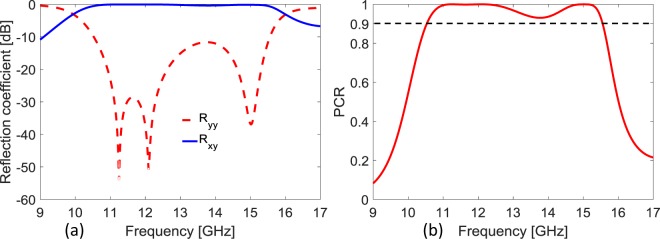
(**a**) Co- and cross-polarized reflection coefficient optimized for *R*_*yy*_ not exceeding −10 dB. (**b**) Polarization conversion ratio (PCR) for the optimized cross-polarizer.

Another interesting aspect of the quasi-crystal metasurface is highlighted in this Section. If we concentrate only on cross-pol efficiency of the subject metasurface, the efficiency can be optimized based on criterion of minimum co-polarized reflection i.e., −10 dB level. In this case, after a comprehensive parametric analysis, the obtained optimized geometric parameters for small/large patches of super cell are *d*_1_ = 1.414 mm, *d*_2_ = 0.707 mm and *r* = 2.85/*R* = 4.2 mm. The optimized thickness of the substrate is *h* =  2 mm. It is emphasized that −10 dB co-polarized reflection is significant as it will ensure high cross-polarization reflection component. Figure 7(a) shows the result of optimized minimum −10 dB level of co-polarization reflection coefficient supporting broadband frequency range 10.5–15.5 GHz.

The low level of co-polarization and high level of cross-polarization directly results into high efficiency of polarization conversion. Polarization conversion ratio (PCR) defines the ratio of reflected power in cross polarized component to total power in both co- and cross- polarized components.

5 $$PCR=\frac{|{R}_{xy}{|}^{2}}{|{R}_{xy}{|}^{2}+|{R}_{yy}{|}^{2}}$$

As depicted in Fig. 7(b), the PCR value for this high-efficiency cross-polarizer remains above 90% over a broad band of 10.5–15.5 GHz with almost 100% PCR from 10.96–12.54 GHz and 14.64–15.29 GHz. It is pertinent to mention here that this optimized cross-polarizer also presents CP operation around 10 GHz (9.8–10 GHz) and 16 GHz (15.89–16.16 GHz) bands.

### Experimental Results

**Figure 8:**
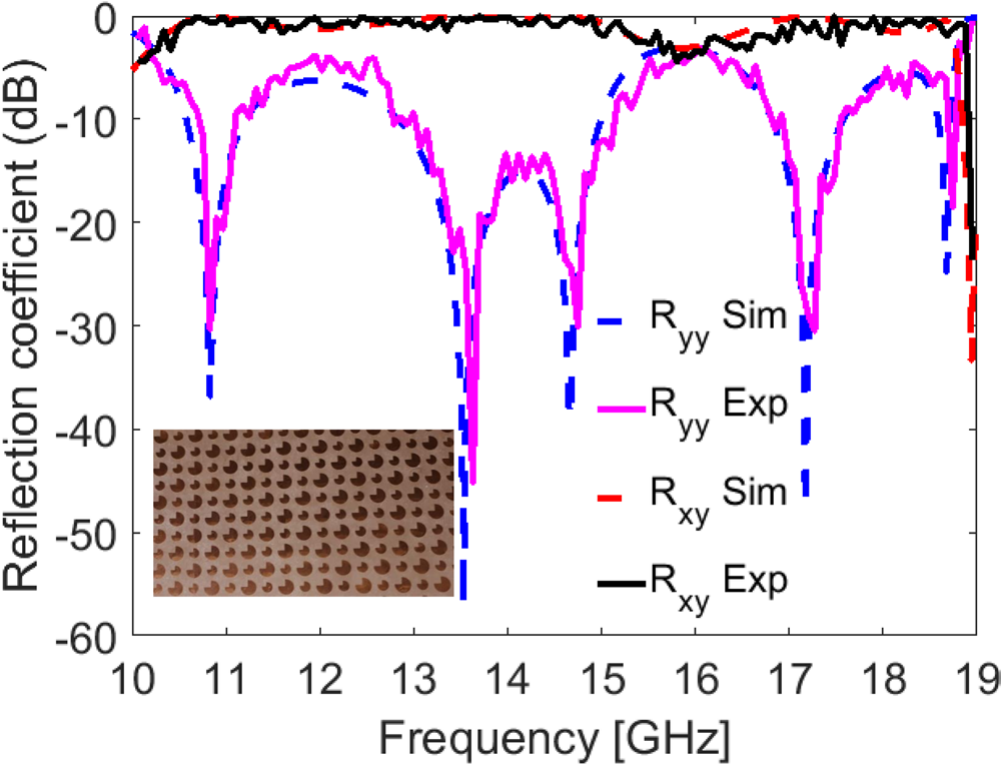
Comparison between experimental and simulation results for the fabricated metasurface shown in the figure inset.

To verify simulation results, the quasi-crystal metasurface is fabricated on Rogers 5880 substrate with cross section of 305 × 228 mm containing 13 × 10 super cell units as shown in the inset of Fig. 8. The experiment was performed in fully anechoic chamber with two broadband horn antennas connected to the vector network analyzer (VNA) for the measurement of reflection coefficients. The transmitting and receiving antennas were placed in vertical direction (*y*- polarized) on the same side in front of the fabricated sample to measure co-polarized reflection *R*_*yy*_. In order to get cross-polarized reflection *R*_*xy*_, receiving horn antenna was rotated in horizontal direction (*x*-polarized). It is clear from Fig. 8 that the simulation results agree well with the experimental results. However, small discrepancies arise due to misalignment of horn antennas and finite cross section of fabricated metasurface.

### Structural Symmetry Analysis

**Figure 9:**
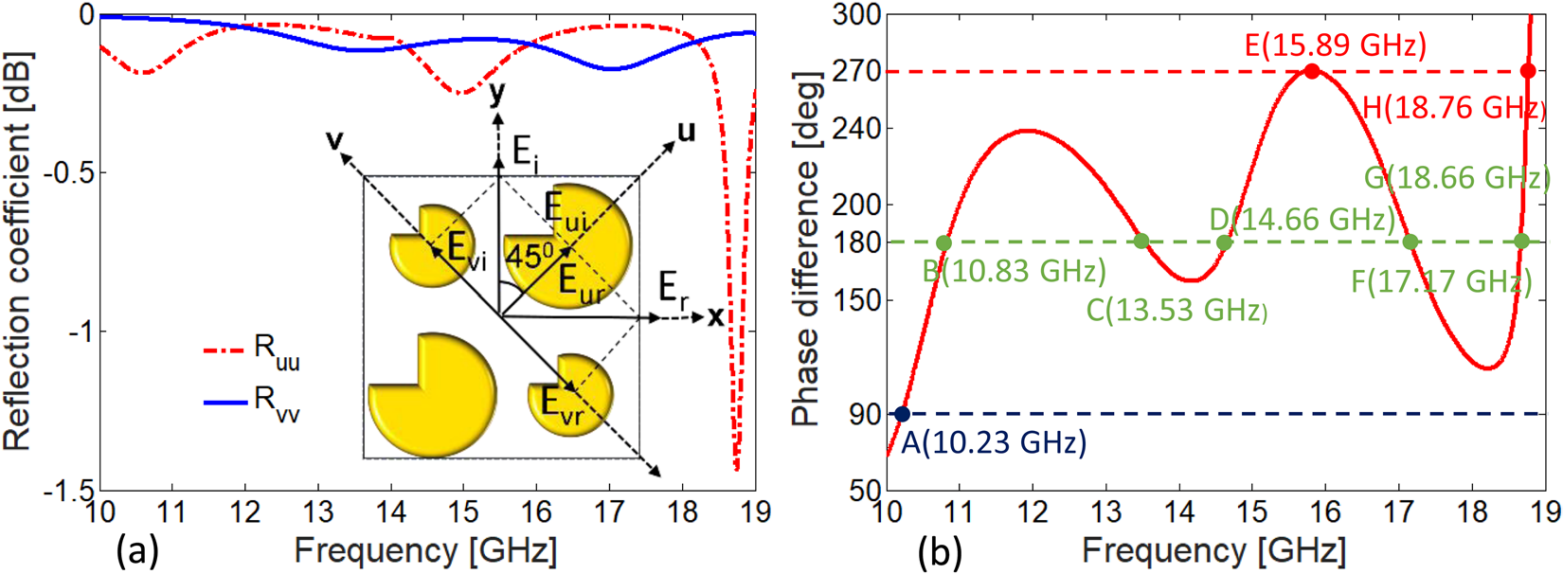
(**a**) Co-polarized reflection coefficient for u- and v- polarized incident waves, the inset shows decomposition of y- axis into u- and v- axes. (**b**) Phase difference between *R*_*uu*_ and *R*_*vv*_.

To better understand the response of the quasi-crystal structure, let us consider that the incident EM wave is polarized along *y*- axis. The incident electric field along *y*- axis can be decomposed into u- and v- components which are ±45 with respect to *y*- axis as shown in Fig. 9(a). It is worth mentioning that the quasi-crystal metasurface exhibits mirror-symmetry around v- axis. The mechanism behind half wave plate and quarter wave plate can be better understood by decomposing the incident and reflected electric fields into u- and v- axes respectively as sketched in Fig. 9(a). We consider *E*_*ui*_ and *E*_*vi*_ as incident electric field components in u- and v- directions respectively. Similarly, consider *E*_*ur*_ and *E*_*vr*_ as reflected electric field along u- and v- directions respectively. The incident electric field in terms of u- and v- axes can be written as follows.

6 $${E}_{i}=u{E}_{ui}+v{E}_{vi}$$

The total reflected electric field can be written as follows.

7 $${E}_{r}=u{R}_{uu}{E}_{ur}+v{R}_{vv}{E}_{vr}$$

Here, *R*_*uu*_ and *R*_*vv*_ represent co-polarized reflection coefficients for u- and v- polarized incident waves respectively. It can be noted in Eqs. 6 and 7 that this formulation to analyze reflected fields along u- and v- axes allows to characterize the response of metasurface avoiding the need to determine cross-polarized components.

The anisotropic nature of quasi-crystal metasurface allows phase difference Δ*ϕ* generated between reflection coefficients i.e., Δ*ϕ* = ∠*R*_*uu*_ − ∠*R*_*vv*_. It should be noted that when the magnitude of reflection components are equal |*R*_*uu*_| = |*R*_*vv*_| and phase difference Δ*ϕ* = ±180°, then the reflected fields will be polarized along ±*x* axis. Recall the incident electric field polarized along *y*- axis, this shows that reflected field polarization is cross-polarized towards *x*-axis direction. It is further emphasized that, when the magnitude of reflection components are equal |*R*_*uu*_| = |*R*_*vv*_| and phase difference Δ*ϕ* = ±90°, then the reflected fields will be circularly polarized.

We analyze the co-polarized reflection coefficients *R*_*uu*_ and *R*_*vv*_ for the quasi-crystal metasurface presented in Fig. 3(a,b). It is evident from Fig. 9(a) that magnitudes of both |*R*_*uu*_| and |*R*_*vv*_| are nearly 1 throughout frequency band. Figure 9(b) shows that the phase difference is nearly Δ*ϕ* = 180° at the five resonance frequencies. Therefore, high efficiency cross-polarization is achieved at the resonance frequencies which are in close agreement with the Fig. 3(a,b). Similarly, circularly polarized reflected wave is obtained at 10.23 GHz, 15.5–16.2 GHz and 18.76 GHz as phase difference of Δ*ϕ* = 90° and 270° is obtained at these frequencies.

### Surface Current Distribution Analysis

**Figure 10:**
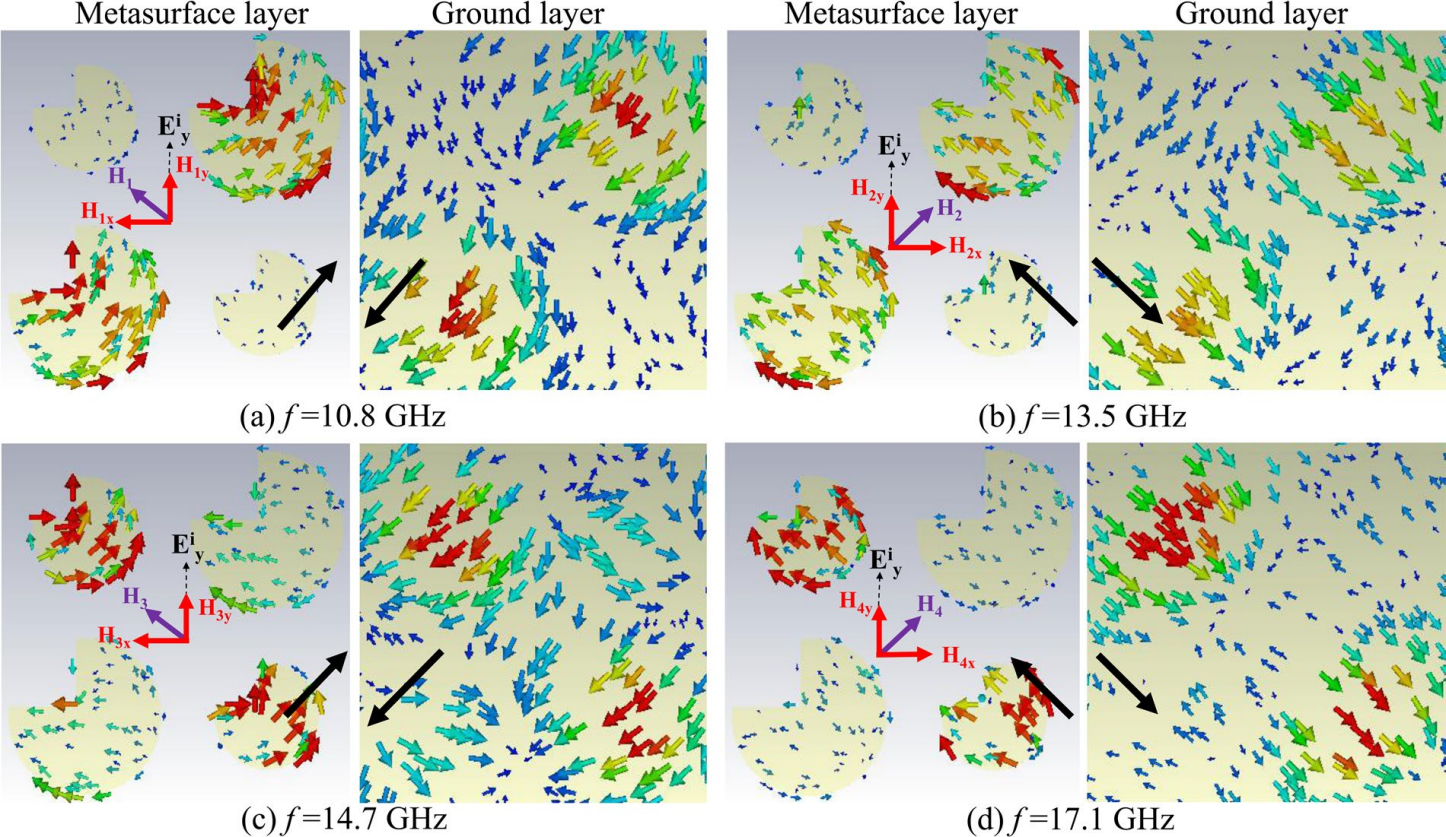
Surface current distributions on quasi-crystal metasurface with response described in Fig. 3b as vector arrows on top metasurface and ground planes at four different resonance frequencies (**a**) 10.8 GHz (**b**) 13.5 GHz (**c**) 14.7 GHz (**d**) 17.1 GHz for *y*- polarized incident wave. The strength and direction of surface current distribution is presented by scaled of size and direction of arrows respectively. The direction of net current in the supercell is given by black arrows at top and bottom layers.

The surface current distribution due to metallic quasi-crystal patches helps to analyze the physical mechanism behind polarization conversion. Figure 10 shows the surface current distributions at the top and bottom ground layers of super cell at various resonant frequencies indicated in Fig. 3(a,b) and described in section 2. The *y*- polarized incident wave will induce coupled surface currents at the top metasurface and ground plane. The direction of these induced currents influence the overall scattering from quasi-crystal metasurface.

It can be noted from the direction of current distribution on both metasurface-plane and ground-plane are anti-parallel to each other, see Fig. 10(a–d). These anti-parallel induced currents at the ground layer with respect to the top layer at the resonance frequency of 10.8 GHz and 14.7 GHz will form closed current loops around the substrate resulting in induced magnetic fields (*H*_1_, *H*_3_) along the v- axis. Cross polarization conversion will not occur due to the *x*-components (*H*_1*x*_, *H*_3*x*_) of induced magnetic field as it is perpendicular to the incident electric field *E*_*iy*_ resulting in no cross coupling. On the other hand, *y*- component (*H*_1*y*_, *H*_3*y*_) of the induced magnetic field at the resonance frequencies is parallel to the incident electric field *E*_*iy*_ resulting in cross coupling and therefore contributes to polarization conversion to *x* axis for the reflected wave.

Similarly, for the magnetic resonance at 13.5 GHz and 17.1 GHz, the induced magnetic fields (*H*_2_, *H*_4_) are parallel to u-axis. Here, the direction having *y*- component (*H*_2*y*_, *H*_4*y*_) of the induced magnetic field is parallel to *E*_*iy*_ thus contributes to *x*-polarized reflected wave. It is interesting to note the current distributions around super cell that shows the larger patch is resonating at 10.8 GHz and 13.5 GHz and smaller patch at 14.7 GHz and 17.1 GHz respectively which is close agreement with Fig. 3.

## Discussion

A design of quasi-crystal metasurface structure is presented to simultaneously work as high-efficiency cross-polarizer and circular polarizer for wide range of frequencies under normal incidence. It is shown that the quasi-crystal metasurface is able to achieve cross-polarization (above −3 dB) at two broad range of frequencies between 10.28–15.50 GHz and 16.21–18.80 GHz. Similarly, the optimized quasi-crystal metasurface is able to suppress co-polarization (below–10 dB) from 10.5–15.5 GHz. In addition, the proposed metasurface is able to efficiently work as high efficiency circular-polarizer from 10.15–10.27 GHz and 15.51–16.20 GHz. Furthermore, the QWP operation of designed metasurface is also angular stable from 0° to 45° in the frequency range of 10.15–10.27 GHz.

## Methods

### The Full-wave Electromagnetic Simulation Method

The unit cell as well as super-cell structures are investigated and analysed through CST Microwave Studio using unit cell boundary conditions along *x*- and *y*- axis. The metallic back plate/ground works as a perfect-reflector to the incident waves having thickness sufficiently larger than the skin depth for incident fields resulting into zero transmission on the other side of the metasurface.

### The Experiment

To practically demonstrate the simulation results, the quasi-crystal metasurface is fabricated on Rogers 5880 substrate with cross section of 305 × 228 mm containing 130 (13 × 10) super cell units. The experiment was performed in fully anechoic chamber with two broadband horn antennas connected to the vector network analyzer (VNA) for the measurement of co- and cross-polarized reflection coefficients. The transmitting and receiving antennas were first placed in vertical direction (*y*- polarized) in front of the metasurface to measure co-polarized reflection coefficient *R*_*yy*_. Then in order to get cross-polarized reflection coefficient *R*_*xy*_, the receiving horn antenna was rotated in horizontal direction (*x*-polarized).

## Data Availability

The datasets generated during and/or analyzed during the current study are available from the corresponding author on reasonable request.
